# TIRF Microscope Image Sequences of Fluorescent IgE-FcεRI Receptor Complexes inside a FcεRI-Centric Synapse in RBL-2H3 Cells

**DOI:** 10.3390/data4030111

**Published:** 2019-07-28

**Authors:** Rachel Drawbond, Kathrin Spendier

**Affiliations:** 1 UCCS Center of the Biofrontiers Institute, University of Colorado at Colorado Springs, Colorado Springs, CO 80918, USA; 2 Department of Mathematics, University of Colorado at Colorado Springs, Colorado Springs, CO 80918, USA; 3 Department of Physics and Energy Science, University of Colorado at Colorado Springs, Colorado Springs, CO 80918, USA

**Keywords:** total internal reflection fluorescence microscopy, TIRF, rat basophilic leukemia cells, RBL-2H3, IgE receptor, FcεRI, plasma membrane, supported lipid bilayer

## Abstract

Total internal reflection fluorescence (TIRF) microscope image sequences are commonly used to study receptors in live cells. The dataset presented herein facilitates the study of the IgE-FcεRI receptor signaling complex (IgE-RC) in rat basophilic leukemia (RBL-2H3) cells coming into contact with a supported lipid bilayer with 25 mol% N-dinitrophenyl-aminocaproyl phosphatidylethanolamine, modeling an immunological synapse. TIRF microscopy was used to image IgE-RCs within this FcεRI-centric synapse by loading RBL-2H3 cells with fluorescent anti-dinitrophenyl (anti-DNP) immunoglobulin E (IgE) in suspension for 24 h. Fluorescent anti-DNP IgE (IgE_488_) concentrations of this suspension increased from 10% to 100% and corresponding non-fluorescent anti-DNP IgE concentrations decreased from 90% to 0%. After the removal of unbound anti-DNP IgE, multiple image sequences were taken for each of these ten conditions. Prior to imaging, anti-DNP IgE-primed RBL-2H3 cells were either kept for a few minutes, for about 30 min, or for about one hour in Hanks buffer. The dataset contains 482 RBL-2H3 model synapse image stacks, dark images to correct for background intensity, and TIRF illumination profile images to correct for non-uniform TIRF illumination. After background subtraction, non-uniform illumination correction, and conversion of pixel units from analog-to-digital units to photo electrons, the average pixel intensity was calculated. The average pixel intensity within FcεRI-centric synapses for all three Hanks buffer conditions increased linearly at a rate of 0.42 ± 0.02 photo electrons per pixel per % IgE_488_ in suspension. RBL-2H3 cell degranulation was tested by detecting β-hexosaminidase activity. Prolonged RBL-2H3 cell exposure to Hanks buffer inhibited exocytosis in RBL-2H3 cells.

## 1. Summary

**Figure 1.**
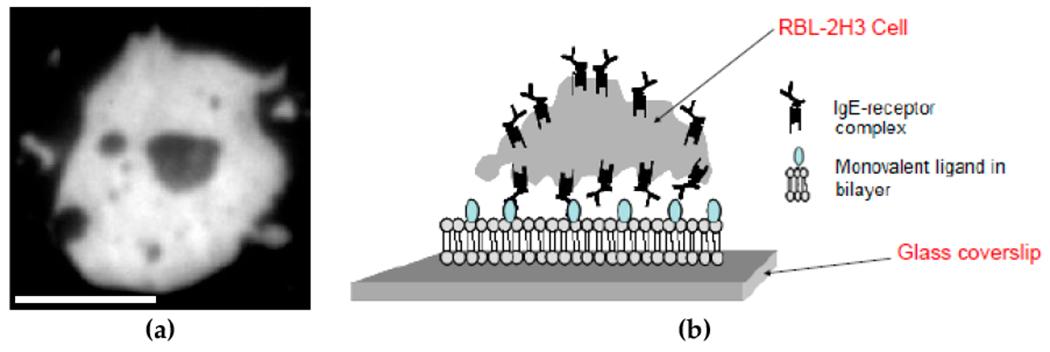
(**a**) TIRF microscope image of RBL-2H3 cell FcεRI-centric synapse. Prior to imaging, the cell was loaded with 80% fluorescent anti-DNP IgE and 20% dark anti-DNP IgE. Scale bar represents 5 μm; (**b**) Schematic of RBL-2H3 cell coming into contact with a supported lipid bilayer with monovalent ligand (25 mol% DNP-lipid) in bilayer.

Understanding cellular transmembrane signaling is critical to almost all aspects of cell biology. Transmembrane signaling plays important roles in cancer and in immune responses, including allergic responses [1–5]. Our principal interest is immune signaling by mast cells. When an antigen, such as a pollen grain, binds multi-valently to immunoglobulin E (IgE)-FcεRI receptor signaling complexes on the cell surface, it causes local aggregation of the receptors, leading to receptor transphosphorylation on their cytoplasmic tails [2,6]. That phosphorylation initiates a well-studied signaling cascade, which ultimately results in the secretion of histamine, serotonin, and other mediators of inflammation. Due to the ability of RBL-2H3 cells to release histamine in an IgE-dependent manner and the expression of high-affinity FcεRI receptors, the RBL-2H3 cell line derived from basophils has been considered to model mast cells and has therefore been used extensively and successfully to study IgE-dependent degranulation [7–10]. To investigate FcεRI receptor aggregation and the formation of the FcεRI-centric synapse, FcεRI receptors on RBL-2H3 cells are typically fully loaded with fluorescently-labeled anti-dinitrophenyl (anti-DNP) IgE and then deposited onto DNP-coated surfaces and observed using total internal reflection fluorescence (TIRF) microscopy [11–15]. TIRF microscopy is widely used to study the motion of cell surface receptors [11,16–25]. Incorporation of DNP-lipid into a bilayer for presentation to cellular receptors provides a mobile ligand. Results show that binding of anti-DNP IgE to mobile DNP results in the large-scale reorganization of receptor clusters to generate the mast cell model synapse or FcεRI-centric synapse shown in Figure 1a [11–13,15]. The ability to form a synapse is a well-known communication strategy between T cells and antigen-presenting cells such as B cells [26,27]. Mast cells have been shown to form a synapse with dendritic cells [28] and γδ T cells [29]. This suggests that mast cells and basophils may play larger roles in signaling between physically contacting cells.

FcεRI receptors on RBL-2H3 cells are typically fully loaded with fluorescent anti-DNP IgE (IgE_488_) when studying the FcεRI-centric synapse [11–15]. This dataset includes images of FcεRI-centric synapses when RBL-2H3 cells are not fully loaded with IgE_488_. In this dataset, RBL-2H3 cells labeled with varying concentrations of fluorescent and dark anti-DNP IgE settled onto supported lipid bilayers (SLBs) with 25 mol% DNP-lipid. Figure 1b depicts a schematic of the experimental model system. IgE_488_ used to label RBL-2H3 cells in suspension 24 h prior to imaging increased from 10% to 100% between datasets, and corresponding non-fluorescent anti-DNP IgE (IgE_dark_) concentrations decreased from 90% to 0%. Prior to imaging, unbound anti-DNP IgE was removed, and primed RBL-2H3 cells were either kept for a few minutes, for about 30 min, or for about one hour in Hanks buffer. Synaptic patches that formed after 15 min in contact with ligand presenting SLBs were imaged using TIRF microscopy. The camera exposure was 5 ms per image, and the pixel size was 107 nm. Additionally, the camera gain was 0.006 photo electrons per analog-to-digital unit (ADU), and the readout noise was 0.529 photo electrons. The fluorescent labeling efficiency of IgE_488_ was 1.02 ± 0.09 mole fluorescent dye per mole protein. The dataset contains dark images to correct for background intensity and TIRF illumination profile images to correct for non-uniform TIRF illumination.

Using dark images and TIRF illumination profile images, image stack pixel intensity values were corrected, and pixel units converted from ADUs to photo electrons. The average pixel intensity within FcεRI-centric synapses for all three Hanks buffer conditions increased linearly at a rate of 0.42 ± 0.02 photo electrons per pixel per percent IgE_488_. Hanks buffer has been widely used over decades as an imaging buffer when RBL-2H3 cells are imaged with a light microscope. Time-dependent effects of the Hanks buffer on the degranulation of RBL-2H3 cells were investigated by measuring extracellular levels of β-hexosaminidase. DNP-bovine serum albumin (BSA) concentration-dependent degranulation of cells that were suspended in Hanks buffer for an hour prior to performing the degranulation assay decreased by 38%-65% compared to cells that were kept in cell media until the degranulation assay was performed. This decrease in degranulation may be an indication that RBL-2H3 cells become stressed when suspended in Hanks buffer for an extended time. Therefore, one further potential research project that could be based on this dataset includes statistical analysis of IgE-FcεRI receptor kinetics when RBL-2H3 cells become stressed. Additionally, the dataset can be used to investigate the concentration fluctuation of the IgE-FcεRI receptor signaling complex within the FcεRI-centric synapse to determining the receptor signaling complex size [30].

## 2. Data Description

**Figure 2.**
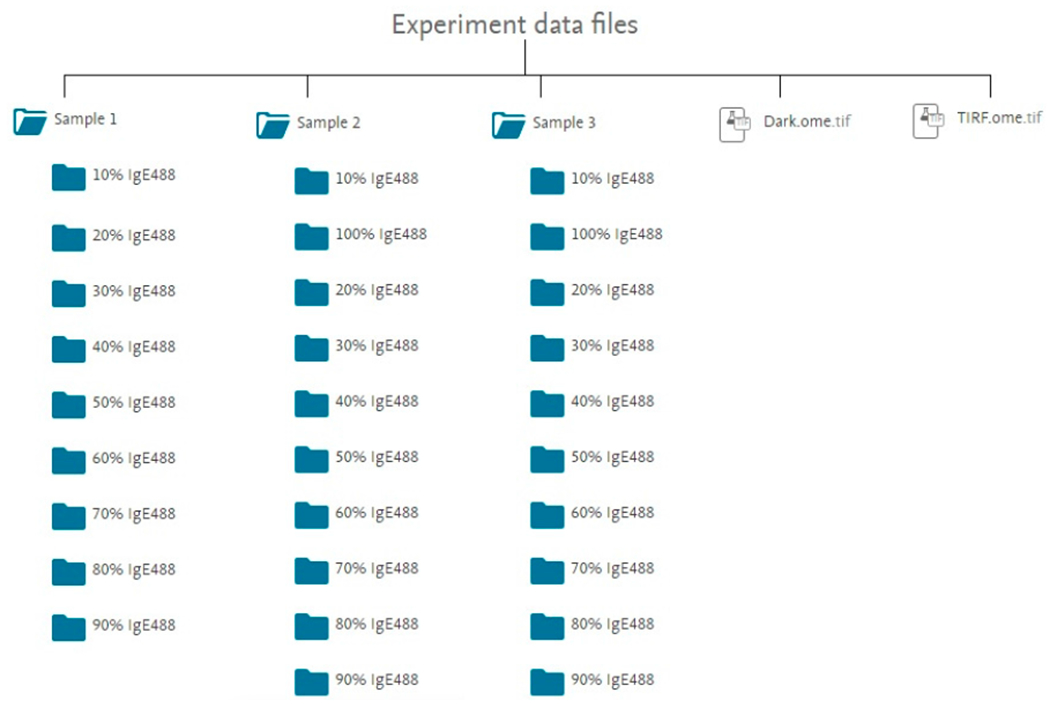
Flowchart of the experiment data files folder and subfolder structure of the published Mendeley data [33]. Cells of Sample 1, Sample 2, and Sample 3 were kept in Hanks buffer for a few minutes, about 30 min, or about 1 h before cells were added to the supported lipid bilayer, respectively. Each subfolder contains between 15 and 26 image stacks of individual RBL-2H3 cell FcεRI-centric synapses saved as ome.tif files as indicated by the number within curved brackets. A dark field image stack (Dark.ome.tif) and a TIRF illumination image stack (TIRF.ome.tif) are also contained in the experimental data files. Each image stack contains a sequence of 500 images.

The data are provided as OME-TIFF (.ome.tif) files, a life sciences file format. Information about the OME-TIFF (.ome.tif) file format as well as imaging software supporting this file format can be found in [31]. The images were captured with an electron multiplying charge-coupled device (EMCCD) camera, Evolve 512 Delta (Photometrics, Tucson, USA) operated by Micro-Manager [32]. The dataset contains 482 RBL-2H3 model synapse image stacks, a dark field image stack (Dark.ome.tif), and a TIRF illumination image stack (TIRF.ome.tif) [33]. All images are 128 × 128 pixel. Each image stack contains 500 individual images with a camera exposure of 5 ms per image and a pixel size of 107 nm. The RBL-2H3 model synapse data is organized into three folders called Sample 1, Sample 2, and Sample 3. Cells of Sample 1, Sample 2, and Sample 3 were kept in Hanks buffer for a few minutes, about 30 min, or about 1 h before cells were added to the supported lipid bilayer, respectively. Each of these folders contains subfolders for ten different fluorescent anti-DNP IgE (IgE_488_) concentrations. IgE_488_ was increased from 10% to 90% by increments of 10% for Sample 1 and increased from 10% to 100% by increments of 10% for Sample 2 and Sample 3. Each subfolder contains between 15 and 26 image stacks, with the majority of subfolders containing 16 image stacks. Each image stack contains 500 image frames of a single RBL-2H3 cell model synapse. Figure 2 shows a flowchart of the data folder structure.

File naming convention for OME-TIFF (.ome.tif) image stacks found in a subfolder is as follows: IgE488_[%]per_Sample[#]_[cell #]_MMStack.ome.tif

**Table:** 

%	refers to the percentage of IgE_488_ and 100% - [%] gives the corresponding percentage of IgE_dark_ for a given image stack.
#	is the sample number. [#] can be 1, 2, or 3.
cell is an integer number ranging from 1 to 26, identifying a particular cell for a given IgE_488_ percentage	
#	and sample number.

**Figure 3.**
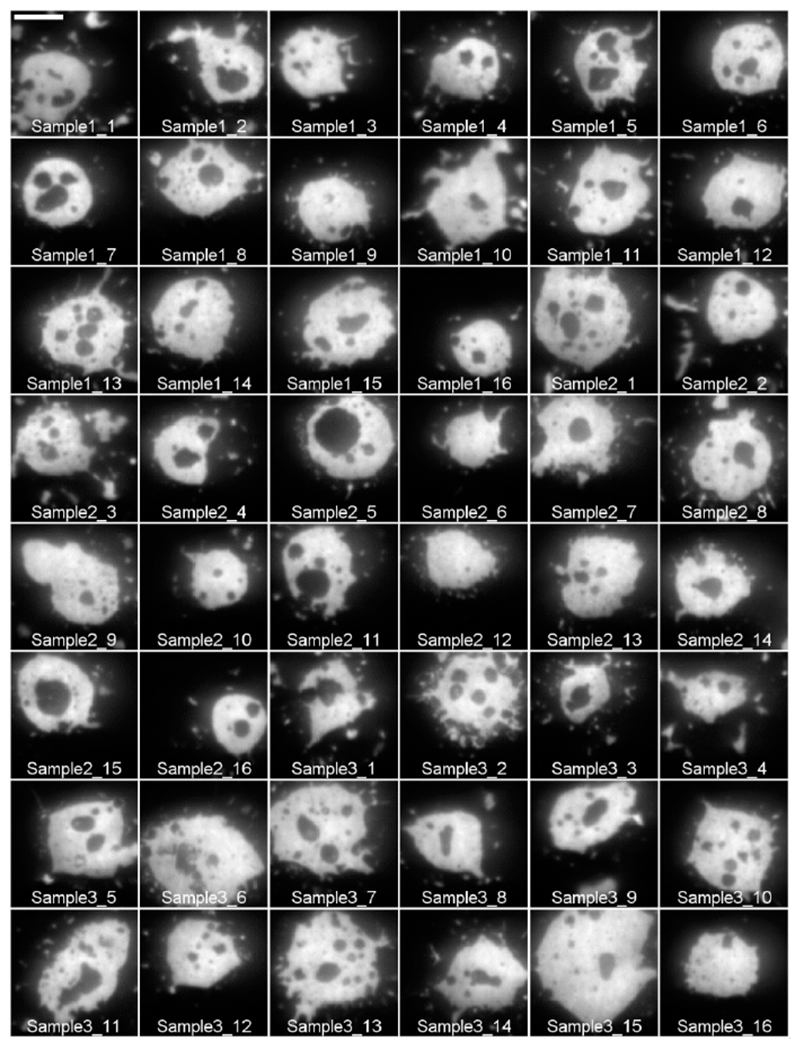
Gallery of images of RBL-2H3 cell model synapses labeled with 80% IgE_488_ and 20% IgE_dark_ in contact with supported lipid bilayers containing mobile ligand. The gallery shows 16 images for each of the three samples. The scale bar in the first image panel represents 5 μm and applies to all remaining panels.

For example, IgE488_80per_Sample1_10_MMStack.ome.tif corresponds to the image stack for cell number 10 in Sample 1 labeled with 80% IgE_488_ and 20% IgE_dark_. Figure 3 depicts a gallery of all RBL-2H3 cell model synapses labeled with 80% IgE_488_ and 20% IgE_dark_ contained in this dataset.

## 3. Methods

This section discusses the methods used to collect the data. First, the RBL-2H3 cell culture is described, and the steps taken to label cells with different percentages of IgE_488_ and IgE_dark_ are outlined. The second section describes the preparation of SLBs, and the third section describes TIRF microscopy with corresponding imaging parameters. The third section describes how camera noise and camera gain were calculated and how the background intensity and TIRF illumination profile image stacks were collected. The last section outlines an IgE-dependent degranulation assay for RBL-2H3 cells.

### 3.1. Labeling of RBL-2H3 Cells at Varying Fluorescent and Dark Anti-DNP IgE Concentrations

The RBL-2H3 cell line was purchased from ATCC (ATTC®, Manassas, USA, CRL-2256). RBL-2H3 cells were maintained in minimal essential medium supplemented with 10% fetal bovine serum, 1% penicillin streptomycin, and 1% L-glutamine. Anti-DNP IgE was purchased from Sigma-Aldrich (~1mg/mL, clone SPE-7, Sigma-Aldrich, St Louis, USA, D8406). Non-fluorescent anti-DNP-IgE (IgE_dark_) was conjugated with 0.15 mM DyLight 488 N-hydroxysuccinimide ester (Thermo Fisher Scientific, Waltham, USA, 46403) according to the manufacturer’s protocols to produce fluorescent anti-DNP-IgE (IgE_488_) and obtain a mole ratio of 1.02 ± 0.08 mole dye per mole protein. A spectrophotometer (NanoDrop™ 2000, Thermo Fisher Scientific, Waltham, USA) was used to measure the absorbance, which was then used to calculate the anti-DNP-IgE concentration and the mole fluorescent dye to mole protein ratio. A concentration of 6.59 ± 0.53 μM or 1.19 ± 0.10 μg/μL was calculated for the IgE_488_ and 7.73 ± 0.26 μM or 1.39 ± 0.05 μg/μL for the IgE_dark_. These concentrations were then used to calculate the volume needed of each to produce a percentage of IgE_488_ in solution that increased from 10% to 100% by increments of 10% and IgE_dark_ in solution that decreased from 90% to 0% by increments of 10%. To label, the calculated volumes of IgE_488_ and IgE_dark_ were added to the RBL-2H3 cells suspended in 10 mL of cell media the day prior to imaging and allowed to incubate overnight (see Table 1). Immediately before experimental data was taken, cells were removed from the suspension dish and resuspended in Hanks buffer. The buffer consists of Hank’s balanced salt solution without MgCl_2_ [34] supplemented with 10 mM 4-(2-hydroxyethyl)-1-piperazineethanesulfonic acid (HEPES) and 0.05% BSA. Cells of Sample 1 were kept in Hanks buffer for a few minutes before cells were added to the SLB. Cells from Sample 2 were kept in Hanks buffer for about half an hour before cells were added to the SLB. Cells from Sample 3 were kept in Hanks buffer for about one hour before cells were added to the SLB.

### 3.2. Supported Lipid Bilayer

Spontaneous liposome fusion was used to produce the SLB [11]. Liposomes composed of 1.3 mM 1-palmitoyl-2-oleoyl-sn-glycero-3-phosphocholine (POPC, Avanti Polar Lipids, Inc., Alabama, USA) and 25 mol% N-dinitrophenyl-aminocaproyl phosphatidylethanolamine (DNP-Cap PE, Avanti Polar Lipids, Inc., Alabama, USA) were made as follows. The chloroform used to suspend the lipids was evaporated under air flow and placed in a vacuum chamber for 1 h to complete the drying process. The lipids were hydrated in 1 mL phosphate buffered saline (PBS, pH 7.4, Fisher BioReagents, Thermo Fisher Scientific, Waltham, USA, BP243820), transferred to a 1.5 mL epitube surrounded by ice and sonicated for 10 min with a probe sonicator. The SLB was prepared on coverslips that had been cleaned prior to the experiment with piranha solution, a mixture of sulfuric acid and hydrogen peroxide. A small amount of liposomes, 50 μL, were placed on a petri dish, and a coverslip was placed over the lipids, which were then allowed to incubate for 15 min at 37 °C to form a laterally mobile bilayer. The coverslip was then transferred to an imaging chamber while submerged under distilled H_2_O. The imaging chamber was washed, multiple times, with 400–500 μL of Hanks buffer before the prepared cells were added. To assure that a central receptor patch was formed before imaging, the cells were allowed to incubate for 15 min on the SLB at 37 °C.

The fluidity of the SLB was tested using a fluorescence recovery after photobleaching (FRAP) method. To do this, liposomes composed of POPC, 25 mol% DNP-Cap-PE, and 0.5% BODIPY-conjugated lipid (Thermo Fisher Scientific, Waltham, USA, D3803) were used to create a SLB. FRAP experiments on the SLB were performed on a confocal microscope (SP5, Leica,, Buffalo Grove, *USA*). The SLB was maintained at 37 °C using an objective heater (Bioptechs, *Butler, USA*). Using the Leica FRAP software, fluorescence recovery curves were fitted to a diffusion model yielding a diffusion coefficient of 1.1 ± 0.3 μm^2^/sec and an immobile fraction of 9.9 ± 7.0%. BODIPY-labeled bilayer uniformity was checked using the widefield mode of the microscope. The bilayer appeared uniform and intact throughout the coverslip surface.

### 3.3. TIRF Microscopy

Objective-based TIRF microscopy of the cells was performed using an S-TIRF module (Spectral Applied Research, Richmond Hill, Canada) attached to a Leica DMI3000B inverted microscope. A 100× and 1.47 numerical aperture oil immersion objective (Leica, Buffalo Grove, USA) in conjunction with a 1.5× tube lens was used for imaging, resulting in an image pixel size of 107 nm. The samples were excited with a 488 nm laser (Coherent Inc., Santa Clara, USA). The penetration depth of the evanescent wave was set to 70 nm. A sample temperature of 37 °C was maintained throughout the imaging process with an objective heater (Bioptechs, Butler, USA). A 525/50 nm single-bandpass filter (Chroma, Bellows Falls, USA) was used to collect fluorescence. The images were captured with an EMCCD camera, Evolve 512 Delta (Photometrics, Yucson, USA) operated by Micro-Manager [32]. The EMCCD camera was set to a multiplier gain of 100. The 128 × 128 pixel region of interest (ROI) allowed for each cell to be imaged with an exposure time of 5 ms.

### 3.4. Calibration and Image Correction

For images contained in this dataset to be useful for further analysis, corrections need to be made to account for background contributions and non-uniform TIRF illumination. Additionally, the collected signal given in analog-digital-units (ADUs) must be converted to photon equivalents or photo electrons using camera gain (g). This signal must then further be corrected by accounting for noise contributions. Noise contributions for EMCCD cameras include Poisson noise, readout noise, dark current noise, and spurious noise. Noise contributions due to the dark current and spurious noise are at least one order of magnitude smaller than typical readout noise contributions and hence are typically ignored [14]. To calculate camera readout noise and gain, a stationary test sample containing the full range of intensities can be used. An appropriate calibration image, which samples all possible intensities, was obtained from out-of-focus fluorescent beads [36]. A time series of 500 images of orange beads (Invitrogen, Thermo Fisher Scientific, Waltham, USA, FluoSpheres® Fluorescent Color Kit, F10720) mounted on a microscope cover glass was taken. Following calibration procedures outlined in references [36] and [13], the camera gain g for this dataset was 0.006 photo electrons per ADU. The readout noise N_read_ was 0.529 photo electrons. To correct for background intensity, 500 images were taken at an exposure of 5 ms with the laser turned off. To correct for non-uniform TIRF illumination, a 1 mm thick, yellow autofluorescent plastic slide (Chroma, Bellows Falls, *USA*) was illuminated in TIRF mode, and an image stack containing 500 images was taken.

### 3.5. Degranulation Assay

**Figure 4.**
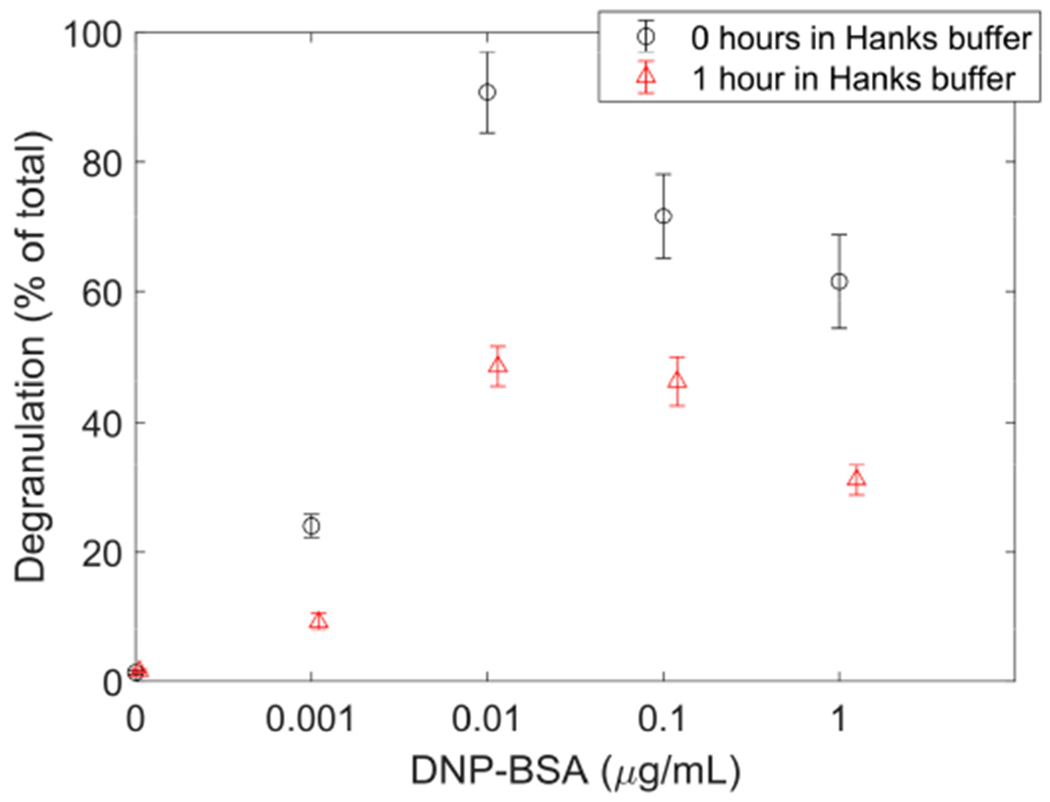
Prolonged RBL-2H3 cell exposure to Hanks buffer inhibits exocytosis in RBL-2H3 cells. Cells were either resuspended for one hour in cell media (black open circles) or in Hanks buffer (red open triangles) prior to the start of a DNP-BSA dose-dependent degranulation assay. Exocytosis or degranulation was measured as the percentage of total cellular hexosaminidase released.

Since the passage number can impact the degranulation process in RBL-2H3 cells, time-dependent effects of Hanks buffer together with concentration-dependent effects of DNP-BSA in solution on the degranulation of RBL-2H3 cells were investigated by measuring extracellular levels of β-hexosaminidase [37]. The degranulation assay was performed on RBL-2H3 cells that were kept in cell media or exposed to Hanks buffer for one hour prior to the start of the degranulation assay. To determine the strength of mast cell degranulation, the amount of β-hexosaminidase, a secreted mast cell enzyme was measured. β-Hexosaminidase, which is generally present in the lysosome, is essential for glycoprotein metabolism in the maintenance of cell homeostasis. In mast cells, large amounts of β-hexosaminidase are present in the granules [38]. In these experiments, 300 × 10^5^ RBL-2H3 cells per well were seeded in two cell culture-treated 24-well plates (ThermoFisher Scientific, Waltham, USA, 142475) and primed for 24 h with 0.5 μg/mL IgE_dark_ in cell media. After 24 h, cells in plate one were washed twice with Hanks buffer and resuspended in a Hanks buffer for one hour. Cells in plate two were also washed twice with Hanks buffer but then resuspended in cell media for one hour. Then the degranulation assay was performed on both plates following the method of Smith et al. [37] with anti-DNP IgE-primed RBL-2H3 cells exposed to 0, 0.001, 0.01, 0.1, or 1.0 μg/mL DNP-BSA (Sigma-Aldrich, St Louis, USA). Supernatants were then collected and the percent of total β-hexosaminidase content released into the medium over the incubation time period were calculated. Figure 4 depicts the release expressed as a percentage of total cellular content for the different experimental conditions. Besides for spontaneous release (0 μg/mL DNP-BSA), the release for cells kept in Hanks buffer instead of cell media for one hour decreased between 38%–65%, depending on DNP-BSA concentrations. The percentage of total cellular hexosaminidase released for 0.1 μg/mL DNP-BSA for cells kept in cell media prior to the start of the degranulation assay is consistent with previous experiments [11,37].

## 4. User Notes

To perform statistical image analysis on this dataset, the following image processing steps should be performed. Image processing is necessary to correct image sequences of the RBL-2H3 cell model synapse for dark current (background intensity), uneven TIRF illumination, and camera noise, as well as to convert original pixel values in ADUs to photo electrons (e^−^). Image processing can be performed in MATLAB in conjunction with DIPimage, an image processing library [39] or with ImageJ, an image processing software freely available in the public domain [40]. First, subtract the dark image D (average of 500 background intensity images) in units of ADU from each image I in units of ADU, and then multiply the image by the TIRF illumination profile T to perform a so-called flat-field correction [41]. To obtain T, compute the average of 500 TIRF illumination images, and then divide this average image by its maximum pixel intensity such that the maximum pixel value of image T is one. Then multiply the resulting image by camera gain g = 0.006 photo electrons/ADU and subtract the readout noise N_read_ = 0.529 photo electrons to obtain the final image S. Equation (1) outlines the calculation to obtain image S. Pixel values of S will be given in photo electrons.

(1) $$\text{S}=\left[\left(\text{I}-\text{D}\right)\text{T}\right]\text{g}-{\text{N}}_{\mathrm{read}}$$

To facilitate the use of this dataset, image stacks to obtain D (Dark.ome.tif) and T (TIRF.ome.tif) are included in the data repository.

**Figure 5.**
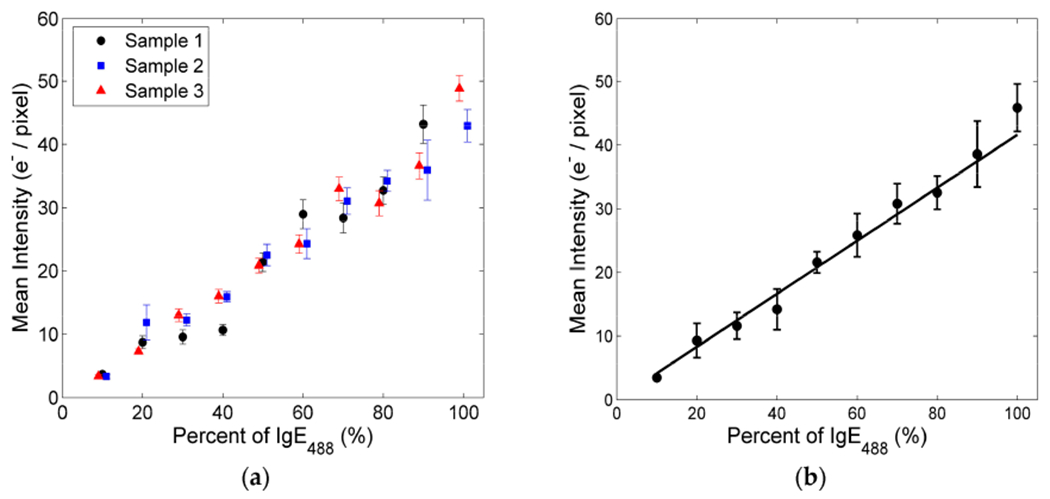
Mean intensity of fluorescently labeled IgE-FcεRI receptor signaling complexes within synaptic patches as a function of percent fluorescent anti-DNP IgE (IgE_488_) added to solution: (**a**) Mean intensity for each sample. *Sample 1* (solid black circles), *Sample 2* (solid blue squares), and *Sample 3* (solid red triangles) were kept in Hanks buffer for a few minutes, about 30 min, or about 1 h before cells were added to the supported lipid bilayer, respectively. Error bars represent the standard deviations; (**b**) Mean intensity of all three samples averaged together with error bars representing the standard deviations. Solid line represents a weighted linear fit *y = a x*. The slope *a* of this fit was 0.42 ± 0.01 photo electrons (e^−^) per pixel per percent (%) IgE_488_ in solution.

It is expected that the concentration of fluorescently labeled IgE-FcεRI receptor signaling complexes within synaptic patches increases linearly with an increasing percentage of IgE_488_. This expected linear trend was verified by comparing the average pixel intensities of imaged FcεRI-centric synapses for different % IgE_488_. The mean fluorescent intensity in units of photo electrons (e^−^) per pixel was calculated by first averaging pixel intensities over individual image stacks after applying Equation (1) and then averaging the intensities of 5–10 square regions (10 × 10 pixels each) within individual FcεRI-centric synapses. Regions within the synaptic patches that contained holes (i.e., no IgE_488_) were not included. Figure 5a shows that the mean pixel intensity inside synaptic patches increased at similar rates as a function of % IgE_488_ for each sample. After averaging pixel intensities for all samples, a line of best fit through the origin was fitted to the data. Figure 5b depicts this fit. The average pixel intensity within FcεRI-centric synapses for all three Hanks buffer conditions increased linearly at a rate of 0.42 ± 0.02 photo electrons per pixel per IgE_488_.

## Figures and Tables

**Table 1.** Volumes of IgE_488_ and IgE_dark_ added to RBL-2H3 cell suspension 24 h prior to imaging and corresponding concentrations for different % IgE_488_ and % IgE_dark_.

% IgE_488_ (%)	% IgE_dark_ (%)	Volume of IgE_488_ Added to Cell Suspension (μL) ^1^	Volume of IgE_dark_ Added to Cell Suspension (μL) ^1^	Concentration of IgE_488_ in Cell Suspension (μ*g*/mL) ^2^	Concentration of IgE_dark_ in Cell Suspension (μ*g*/mL) ^2^
10	90	0.5 ± 0.1	3.8 ± 0.1	0.06 ± 0.1	0.53 ± 0.3
20	80	1.0 ± 0.1	3.4 ± 0.1	0.12 ± 0.2	0.47 ± 0.2
30	70	1.5 ± 0.1	3.0 ± 0.1	0.18 ± 0.2	0.41 ± 0.2
40	60	2.0 ± 0.1	2.6 ± 0.1	0.24 ± 0.2	0.36 ± 0.2
50	50	2.5 ± 0.1	2.1 ± 0.1	0.30 ± 0.3	0.30 ± 0.2
60	40	3.0 ± 0.1	1.7 ± 0.1	0.36 ± 0.3	0.24 ± 0.2
70	30	3.5 ± 0.1	1.3 ± 0.1	0.42 ± 0.4	0.18 ± 0.2
80	20	4.0 ± 0.1	0.9 ± 0.1	0.48 ± 0.4	0.12 ± 0.1
90	10	4.5 ± 0.1	0.4 ± 0.1	0.54 ± 0.5	0.06 ± 0.1
100	0	5.0 ± 0.1	0.0	0.60 ± 0.5	0.00

1 Pipetting errors using a micropipette.

2 Errors obtained from basic propagation of errors rules [35], i.e., *x* and *y* have errors *δx* and *δy*, then the error in *z* = *x* × *y* is $\frac{\delta z}{z}=\sqrt{{\left(\frac{\delta x}{x}\right)}^{2}+{\left(\frac{\delta y}{y}\right)}^{2}}$
